# Medication Errors in Psychiatric Hospitals: A Nationwide Real-World Evidence Study in Saudi Arabia

**DOI:** 10.3390/ph17111514

**Published:** 2024-11-11

**Authors:** Khalidah A. Alenzi, Mona Y. Alsheikh, Deemah S. Alsuhaibani, Yasser Alatawi, Thamir M. Alshammari

**Affiliations:** 1 Transformation, Planning and Business Development, Tabuk Health Cluster, Tabuk 47717, Saudi Arabia; ph_kh@hotmail.com; 2 Society of Pharmacovigilance, Jeddah 23434, Saudi Arabia; 3 Pharmacy Practice Department, Faculty of Pharmacy, King Abdulaziz University, Jeddah 22254, Saudi Arabia; myalsheikh@kau.edu.sa; 4 Pharmaceutical Care Department, Medical Services for Armed Forces, Ministry of Defense, Riyadh 12626, Saudi Arabia; deemahshb@gmail.com; 5 Department of Pharmacy Practice, Faculty of Pharmacy, University of Tabuk, Tabuk 47512, Saudi Arabia; yasser@ut.edu.sa; 6 Department of Clinical Practice, College of Pharmacy, Jazan University, Jazan 45142, Saudi Arabia; 7 Pharmacy Practice Research Unit, College of Pharmacy, Jazan University, Jazan 45142, Saudi Arabia

**Keywords:** medication error, primary care centers, psychiatric medications, Saudi Arabia

## Abstract

**Background**: Medication errors are among the most prevalent medical errors and result in significant morbidity and mortality. They pose a major threat to patient safety in psychiatric hospitals. However, the lack of a comprehensive investigation into the root causes of these errors restricts the development of effective corrective measures. **Objective**: This study aimed to characterize the types of errors, determine the stages of the medication use process, and identify factors associated with errors occurring among government psychiatric hospitals in Saudi Arabia. **Methods**: This cross-sectional study was conducted from August 2019 to June 2020. All medication error reports submitted to the Ministry of Health General Administration of Pharmaceutical Care database from 18 government psychiatric hospitals in Saudi Arabia were assessed. The database is de-identified and contains information on all medication errors, including patient demographics, medication information, error information, causes of errors, and reporter information. Medication use was categorized as ordering/prescribing, transcribing, dispensing, administration, and monitoring. The present findings represent a descriptive analysis of the data using Statistical Analysis Software (SAS) version 9.4. **Result:** A total of 23,355 medication error reports were reported to the database during the study period. Among Saudi Arabian cities, Riyadh (*n* = 8972, 38.4%) had the highest medication error reporting rate, followed by Taif (*n* = 3705, 15.9%) and Jeddah (*n* = 2621, 11.2%). Most reported errors were frequently made by physicians (*n* = 20,284, 86.9%) and were primarily detected by pharmacists (*n* = 20,974, 89.8%). Approximately half of them (*n* = 13,150, 56.3%) were classified as “Category B” that occurred, but they did not reach patients. Most medication errors were reported in adults (*n* = 22,589, 96.7%) and male patients (n = 16,393, 70.2%). Most error reports were detected at the prescription stage (n = 16,481; 70.6%) during the medication-use process. Work overload of the staff (*n* = 2911, 12.8%) and drug labeling, packaging, and nomenclature (*n* = 2826, 12.1%) were the most common contributing factors associated with the reported errors. Olanzapine (*n* = 1650, 7.1%), omeprazole (*n* = 1350, 5.8%), and quetiapine (*n* = 1273, 5.5%) were the most common medications associated with medication errors. **Conclusions:** Preventable medication errors are typical in psychiatric hospitals and may result in severe consequences. Increased efforts are needed to control and minimize prescribing errors and improve reporting in Saudi Arabia.

## 1. Introduction

Historically, hospitals and clinics in Saudi Arabia have had a limited capacity to hospitalize psychiatric patients. The Shehar Mental Hospital in Taif, the first psychiatric hospital, opened in 1952 and had a capacity of 250 beds [1]. Mental health services in Saudi Arabia have existed and evolved over time. Currently, 21 mental health hospitals with a capacity of 4046 beds and 99 psychiatric clinics are operational, and 14 specialized hospitals are under construction [2]. Globally, 32% of years lived with disability (YLDs) and 13% of disability-adjusted life years (DALYs) are due to mental illnesses [3]; despite the prevalence of these disorders, the quality of care for these conditions remains substandard. Persistent gaps in access to mental health services exist [4].

Patients with psychiatric diseases primarily present multiple comorbidities that require treatment with non-psychiatric medications. Unfortunately, when these patients are treated in mental hospitals, a higher risk of medication errors (MEs) exists due to the poor quality of medical care [5,6]. The findings of the Japanese Adverse Drug Event study (JADE) corroborated those from the study by Keer, indicating that the percentage of preventable adverse drug events associated with non-psychiatric drugs was three times more than that of psychiatric drugs, at 36% to 13%, respectively [6].

MEs have received considerable attention in recent years because they are associated with substantial mortality, morbidity, and additional healthcare costs. Despite extensive research aimed at understanding the frequency, causes, and preventive strategies for MEs in general hospital settings, mental health hospitals have received less attention [7]. The retrospective study by Grasso et al., examining the incidence of MEs in psychiatric inpatients, concluded that 58% of MEs were assessed as having a high risk of harm [8].

MEs are generally divided according to the phase of the process at which the error arises, subdivided into errors of prescribing, transcription, dispensing, monitoring, and administration or classified based on assumptions regarding their causes [9]. A study conducted in Denmark revealed that the most common MEs occurred during administration (75%), dispensing (10%), and prescribing (5%) [10].

This study aimed to thoroughly investigate medication errors (MEs) in psychiatric hospitals, addressing a range of conditions, both psychiatric and chronic diseases. The research focused on understanding the severity of these errors and assessing how they impact patient safety and treatment outcomes. Additionally, the study sought to pinpoint the specific stages of the frequency of errors and medication process—such as prescribing, dispensing, administering, and monitoring—where these errors typically arise, providing a comprehensive overview of the challenges in medication management within these healthcare settings.

## 2. Results

### 2.1. Demographic Characteristics

**Figure 1:**
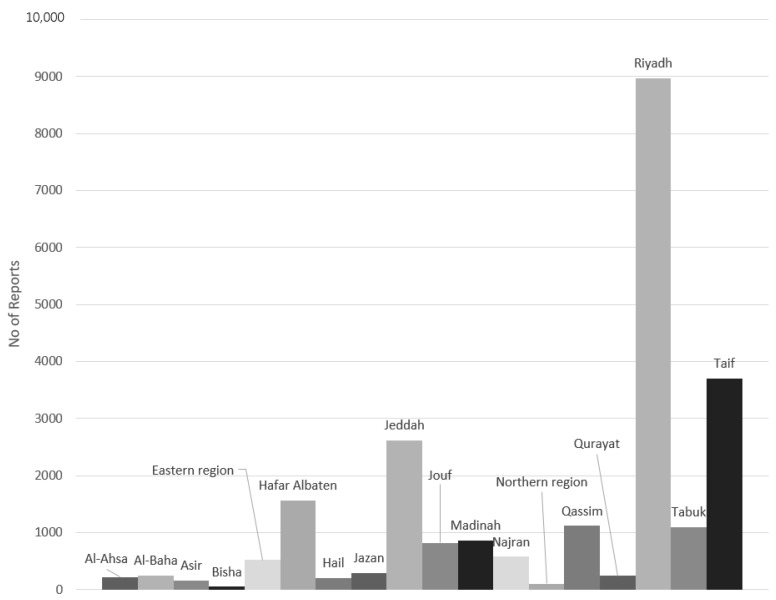
Frequency distribution of medication error reports according to cities in Saudi Arabia.

From August 2019 to June 2020, 23,355 ME reports from 18 governmental mental health and psychiatric hospitals in Saudi Arabia were submitted to the MOH database. The highest rate of reported MEs was from Riyadh (*n =* 8972; 38.42%), followed by Taif (*n* = 3705; 15.86%), Jeddah (*n* = 2621; 11.2%), and Hafar Albaten (*n =* 1565; 6.70) (Figure 1).

Most MEs were reported in male patients (*n* = 16,393; 70.19%) and adults (*n* = 22,589; 96.72%). The majority of the MEs were committed by physicians (*n =* 20,284; 86.85%), followed by pharmacists (*n* = 1824; 7.81%), and nurses (*n* = 861; 3.69%); on the other hand, these MEs were primarily detected by pharmacists (*n* = 20,974; 89.81%), followed by nurses (*n* = 1464; 6.27%), and physicians (*n* = 428; 1.83%). More than half of the reported MEs were classified as “Category B” (*n* = 13,150; 56.30%), followed by “Category A” (*n* = 7344; 31.45%), and “Category D” (*n* = 1741; 7.45%). Most MEs were detected at the prescription stage (*n* = 16,481; 70.57%), followed by the preparation stage (*n* = 1702; 7.29%) and the administration stage (*n* = 1576; 6.75%) of the medication-use process (Table 1).

### 2.2. Factors Contributing to MEs

**Figure 2:**
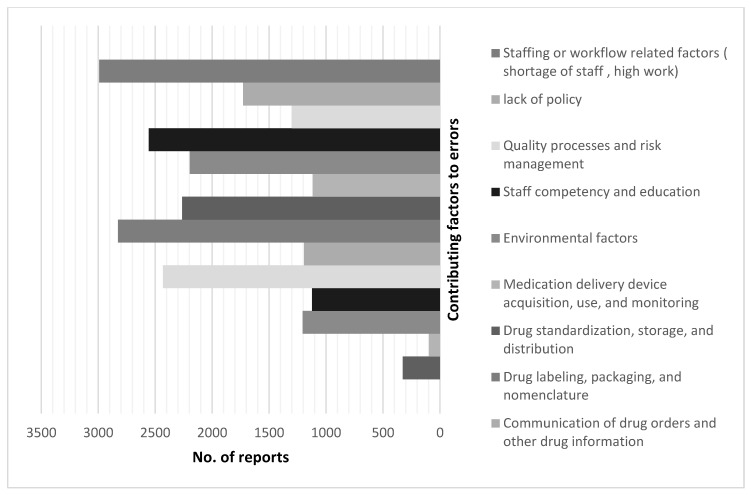
Factors contributing to medication errors.

The workload of healthcare staff (*n* = 2991; 12.81%) drug labeling, packaging, and nomenclature (*n* = 2826; 12.10%) were the most common contributing factors associated with the reported MEs, followed by healthcare staff competency and education (*n* = 2557; 10.95%) and patient information (*n* = 2432; 10.41%) (Figure 2).

### 2.3. Therapeutic Categories Associated with MEs

**Figure 3:**
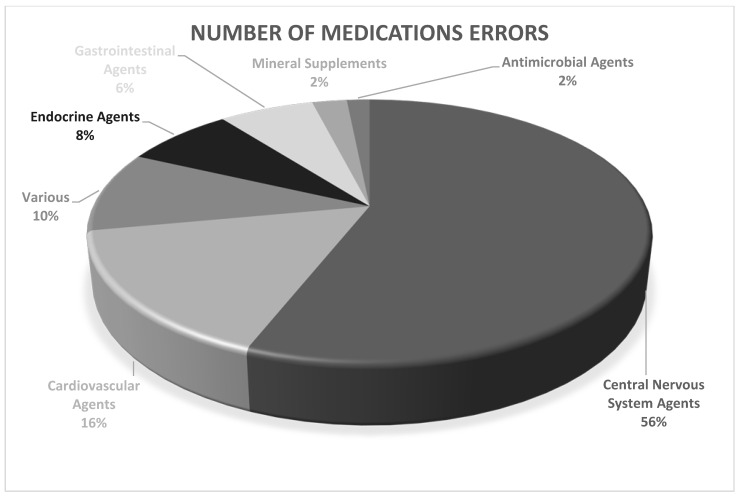
Reported medication errors classified by USP therapeutic categories.

More than half of the reported MEs in the USP therapeutic category were associated with central nervous system (CNS) agents, which accounted for 13,076 cases (55.99%). Cardiovascular agents were the second most common with 2319 cases (16.04%). Among CNS agents, antipsychotics were the most frequent cause of MEs with 7769 cases (59.41%), followed by antidepressants with 3829 cases (29.28%), anticonvulsants with 750 cases (5.74%), and sedatives/hypnotics with 607 cases (4.64%). (Table 2 and Figure 3).

### 2.4. Percentage of Reported MEs for the Top Twenty Medications

Among 22,355 ME reports, olanzapine (*n* = 1650; 7.06%), omeprazole (*n* = 1350; 5.78%), quetiapine (*n* = 1273; 5.45%), mirtazapine (*n* = 1120; 4.80%), haloperidol (*n* = 1045; 4.47%), and risperidone (*n* = 1000; 4.28%) had the highest percentages of reported MEs among the top 20 medications (Table 3).

Additionally, labetalol and insulin aspart were associated with errors, which may have contributed to or resulted in temporary harm and hospitalization.

Our study has several limitations. First, it could not differentiate whether the detected errors were the same as or different from the original errors. Second, the ME report did not elaborate on the contributing factors in detail, such as staffing—or workflow-related factors. However, our study is strengthened by being the first to report on MEs in psychiatric hospitals nationally, representing all regions of Saudi Arabia.

## 3. Discussion

MEs are common, often preventable, and have a substantial impact on patient health, especially those that occur in psychiatric hospitals. However, few studies have reported MEs in these hospitals. Therefore, this study provides real-world data on such errors.

We studied these errors in all mental health hospitals with different clinical capacities, and the presence of addiction centers in all regions of the Kingdom caused discrepancies in documenting the errors. We found the most reports from Riyadh psychiatric hospitals, with 700 beds, followed by Shahar Hospital in Taif, with 670 beds, reflecting our results [2].

In line with our findings, a study conducted at Japanese psychiatric hospitals revealed that approximately 53% of men in long-term psychiatric care units were affected by medication errors [11].

In a systematic review, Maidment et al. explored the incidence and causes of MEs in psychiatric hospitals. The most frequently reported and detected errors by pharmacists during routine prescription examinations were identified in the study. Moreover, physicians had the lowest reporting rates for MEs, similar to our findings [12]. Another study in Saudi Arabia revealed that physicians made 88.5% of MEs, and most were detected by pharmacists (75.9%) [9]. Also, a study conducted at a French psychiatric hospital (2014–2021) found that 609 medication errors were recorded, with 61.4% detected by pharmacy staff [13]. 

The reason for pharmacists’ frequent discovery of medication errors is related to encouraging the general administration of pharmaceutical care at the Saudi MOH by using key performance indicators to enhance the reporting system.

The same study in Saudi Arabia revealed that 66.3% of errors occurred and reached the patient but did not cause harm: (Category B), followed by Category A (21%). In contrast, 6.8% of errors reached the patient and were associated with harm. These results are identical to those from our study [9]. 

Likewise, a retrospective, cross-sectional study conducted over eight years at a French psychiatric hospital (2014–2021) found that out of 253 medication errors (MEs), none resulted in serious harm to patients (Categories C and D). Additionally, 2.1% of the errors led to significant adverse reactions (Categories G and H), while three cases resulted in death [13].

Our results show that most medication errors occurred during the prescription stage, followed by the administration stage. This is consistent with findings from another comprehensive review, which indicated that prescription errors ranged from 76.3% to 87.5% [12]. Another study found that 42% of errors were identified during the administration stage [10]. Meanwhile, the study by Ayana indicated that the monitoring and ordering stages were most associated with errors (39% and 34%, respectively) [6].

In 2006, the first comprehensive analysis of medication safety incidents—including medication errors (MEs) and adverse drug events (ADEs)—was published for mental health trusts in England and Wales. This analysis used data from the National Reporting and Learning System (NRLS) database and documented 1648 medication incidents reported to the NRLS between 2003 and September 2005. These incidents represented 3.7% of the total recorded patient safety incidents. A significant majority, precisely 64.6% (1065 incidents), occurred during the medication administration stage. Furthermore, 12.6% (208 incidents) were linked to the prescribing phase, while 12.3% (202 incidents) pertained to medication preparation and dispensing. The three most frequently reported categories of medication incidents were wrong or unclear doses, accounting for 20.1% (332 incidents); wrong drugs, representing 14.0% (231 incidents); and incorrect frequency, comprising 12.8% (221 incidents) [14]. 

Understanding medication errors (MEs) in psychiatric hospitals is limited due to several factors related to mental health and patients’ clinical conditions. These factors include forced medication administration, which may lead to patients resisting treatment. Additionally, patients might misunderstand or fail to follow healthcare professionals’ advice, resulting in medication errors. The complexity of the medications used for psychiatric disorders, along with limited clinical pharmacy services, further complicates the situation [6,15].

Another important factor is that patients with mental health issues (MHIs) may be transferred to various healthcare settings, including mental health hospitals, acute hospitals, primary care, and community healthcare services. These transfers can be particularly risky, as patients might experience changes in their health status and prescribed medications during this time. As a result, a lack of coordination between these settings can put patients at risk of harm and may lead to them moving between facilities without receiving appropriate care [16]. Data from the National Reporting and Learning System (NRLS) revealed that 33% of 10,000 reported incidents occurred during the discharge process from secondary mental health care to either primary or community care. A significant factor contributing to these incidents was the miscommunication of patient medication information. In response, NHS England issued a patient safety alert to aid mental health care organizations in enhancing the quality of information transfer during discharge. The alert recommends implementing medication reconciliation to mitigate the risk of such incidents [17].

Dean et al. define prescribing errors as “a clinically meaningful prescribing error that occurs when, as a result of a prescribing decision or prescription writing process, there is an unintentional significant reduction in the probability of treatment being timely and effective or an increase in the risk of harm when compared with generally accepted practice.” [18]. In a comprehensive review of MEs in psychiatry hospitals, Procyshyn et al. revealed that the two most common factors causing prescription errors were a lack of knowledge of medications and patients’ clinical status [19]. Two studies conducted in the UK found that senior physicians were more likely than junior physicians to make prescribing errors (PEs). Additionally, using an electronic prescription template and increasing the number of medication items prescribed raised the risk of PEs in discharge prescriptions [7,20]. 

However, the study conducted by Sorensen et al. (2013), observing 1082 cases of drug administration, reported 189 errors and indicated that the most common contributing factors were related to the skills of the nurses in administrating medication (75%), followed by incorrect prescriptions (10%), and illegibility and incomprehensibility of the writing of physicians (5%) [10]. 

Nevertheless, a study conducted by the National Health Service in England revealed that most MEs made by nurses were skill-based. Other factors included inadequate staffing levels, an unbalanced staff skill mix, interruptions and distractions, drug-related factors, and communication [5]. In our study, staff work overload and drug-related factors, such as labeling and packaging, followed by staff competency and education, were the most common contributing factors.

We show that psychiatric drugs were responsible for most MEs, and non-psychiatric drugs, such as cardiovascular and endocrine drugs, caused nearly a quarter of the MEs, which may be due to the lack of knowledge of the psychiatrists, lack of familiarity with these agents, and specific complexities of the medications used to treat psychiatric patients. According to the results of the study by Ayani et al., non-psychiatric medications are three times more likely to cause errors than psychiatric drugs [6,21].

A study conducted between 2010 and 2017 reported 94,134 medication incidents from inpatient mental health settings across England and Wales to the National Reporting and Learning System (NRLS). Central nervous system (CNS) medications accounted for the majority of these reports, representing 68.4% of incidents with a total of 42,609 cases. Within this category, antipsychotics were the most commonly involved drugs, constituting 35.0% of reports, followed by anxiolytics and hypnotics at 19.1% and antidepressants at 13.5%. The incidence of psychotropic medications was comparable to that of non-psychotropic medications, at 49.1% and 50.8%, respectively. Other frequently reported medication categories included cardio-vascular drugs (7.2%), endocrine medications (5.8%), and treatments for infectious diseases (4.4%). A limited number of severe incidents were associated with immunosuppressants (3.7%), anesthetics (3.1%), and endocrine medications (2.2%). Notably, these specific classes demonstrated a higher proportion of incidents resulting in severe outcomes when compared to other medication classes [22].

In our study, Labetalol and Insulin Aspart were associated with harmful errors associated with hospitalization. These errors may be related to the reasons discussed previously, and when cross-matched with contributing factors, they cause these errors. Our findings indicate that MEs are associated with staffing—or workflow-related factors and drug standardization, storage, and distribution. This suggests that the staff, even prescribing nurses or pharmacists, are unfamiliar with handling such medications.

A retrospective analysis conducted using data from the National Poison Data System revealed that the medications most frequently associated with errors were antidepressants (70.1%) and antipsychotics (29.9%). Selective serotonin reuptake inhibitors were identified as the most common drug associated with errors (30.3%), followed by atypical antipsychotics (24.1%) and other antidepressants (21.5%) [23]. A report published in 2006 by mental health trusts in England and Wales utilized data from the NRLS database. It revealed that antipsychotics were the most involved medication class in reported incidents, accounting for 42.6% of medication-related incidents. This was followed by anxiolytics at 20.0% (111 incidents), antidepressants at 13.3% (74 incidents), and analgesics at 11.7% (65 incidents) [14]. These results support our finding that atypical antipsychotics, such as Olanzapine and quetiapine, are the most commonly associated with MEs because they may be prescribed more frequently in psychiatric hospitals. Other rationales for the errors may be related to the patent expirations of the atypical antipsychotic drugs, such as Olanzapine (Zyprexa^®^), Risperidone (Risperdal^®)^, and Quetiapine (Seroquel^®^). This may enable the manufacture of generics with different packaging or labeling, increasing the possibility of mistakes [23].

Finally, our understanding of medication errors (MEs) in psychiatric hospitals is less developed than in other specialized healthcare sectors. The healthcare system should implement several key initiatives to reduce these errors significantly. These include mandating medication reconciliation programs, effectively utilizing applied clinical pharmacy roles, standardizing medication dosage forms, and establishing Electronic Prescribing and Medicines Administration (EPMA) systems. Additionally, implementing pharmacist-led Information Technology Interventions for Medication Errors (PINCER) and developing robust ME reporting systems can further enhance patient safety. In addition, the implementation of medication reconciliation and continuing education programs, especially for non-psychiatric medication, should be promoted for all healthcare providers, and it should also be ensured that a specialist prescribes these medications.

## 4. Materials and Methods

### 4.1. Study Design and Data Source

This retrospective observational study involved the analysis of electronic medication error reports sourced from 18 government mental health and psychiatric hospitals located across Saudi Arabia. The data utilized for this study were obtained from the General Administration of Pharmaceutical Care Database, covering the period from August 2019 to June 2020. It is worth noting that this database is developed and maintained by the Ministry of Health (MOH) and serves as a central repository for all reports from regional medication safety and quality management committees. Furthermore, the MOH provides regular monthly updates detailing the quality and quantity of medication error reports, thereby contributing to the ongoing monitoring and assessment of medication safety practices.

In a hospital setting, the procedure for reporting medication errors by medical staff involves the following steps: completing a paper-based reporting form and submitting it to the medication safety officer within 24 h. Then, the medication safety committee assesses the event and conducts a root cause analysis. After that, a medication safety officer submits the error on the General Department of Pharmaceutical Care website of the Ministry of Health.

The ME reports underwent de-identification, and any duplicate reports identified in the dataset were removed. These reports were then classified based on various parameters such as sex (male or female), age (in years) at the time of the report, date of error, contributing factors, sources, and detection of medication errors. Furthermore, the ME reports were categorized according to patient outcomes, and detailed stages of the medication use process were recorded. Moreover, medications were classified following the guidelines outlined in the United States Pharmacopeia (USP) Therapeutic Category Model [24].

### 4.2. ME Categories

The United States National Coordinating Council (NCC) for Medication Error Reporting and Prevention (MERP) definition and patient outcome categories of MEs were used to identify the errors. The NCC MERP defines an ME as “Any preventable event that may cause or lead to inappropriate medication use or patient harm while the medication is in the control of the health care professional, patient, or consumer.” [25]. The NCC MERP categorizes patient outcomes of MEs into nine categories, ranging from categories “A” to “I”. Category “A” represents circumstances or events that can cause an error without reaching the patient. Category B represents an error that occurred, but the medication did not reach the patient; Categories “C” and “D” represent errors that reached the patient but did not cause harm. Specifically, Category D represents an error that resulted in the need for increased patient monitoring but no patient harm. Categories “E”, “F”, “G”, and “H” represent errors that resulted in patient harm. Specifically, Category E represents an error that resulted in the need for treatment or intervention and caused temporary patient harm, Category F represents an error that resulted in initial or prolonged hospitalization and caused temporary patient harm, and Category G represents an error that resulted in permanent patient harm. Category H represents an error that resulted in a near-death event. Category “I” represents an error linked to the patient’s death [25].

### 4.3. Government Mental Health and Psychiatric Hospitals

The study involved 18 governmental mental health and psychiatric hospitals in Saudi Arabian cities, which are Al-Ahsa, Al-Baha, Al-Jouf, Asir, Al-Gassim, Al-Qurayat, Al-Madinah, Bisha, Eastern Region, Hafar Albaten, Hail, Jazan, Jeddah, Najran, Northern Region, Riyadh, Taif, and Tabuk.

### 4.4. Statistical Analyses

Descriptive statistics were presented as frequencies (n) and percentages (%). Data cleaning and management were performed using Microsoft Excel (2016 Version; Redmond, WA, USA).

Data are presented as frequencies and percentages. The overall rate of MEs was calculated by dividing the number of reported errors by the total number of prescriptions. In addition, the data included age (grouped as >1 year–13 years, >13 years–18 years, and >18 years). The descriptive data were analyzed using the Statistical Analysis Software (SAS^®^ 9.4, Cary, NC, USA).

## 5. Conclusions

Medication errors are of great concern in the medications that are used in psychiatry hospitals and require greater attention from stakeholders. These errors were associated with psychiatric medications as well as non-psychiatric medications that require increased attention. Most of these errors resulted from the lack of skills among the health staff, an increase in workload, and a lack of standardization of dosage forms. However, further efforts by regulators and health policymakers are needed to reduce MEs in Saudi Arabia, especially in the field of psychiatry.

## Figures and Tables

**Table 1:** Descriptive characteristics of the medication error reports.

Characteristics	Frequency (n)	Percentage (%)
TOTAL (*n*) = 23,355		
Sex		
Male	16,393	70.19%
Female	6360	27.23%
Not Specified	602	2.58%
Age		
Child (>1–13 years)	59	0.25%
Adolescent (>13–18 years)	56	0.24%
Adult (>18 years)	22,589	96.72%
Not Specified	651	2.79%
Sources of Medication Errors		
Physician	20,284	86.85%
Pharmacist	1824	7.81%
Nurse	861	3.69%
Patient	352	1.51%
Other	34	0.15%
Detection of Medication Errors		
Pharmacist	20,974	89.81%
Nurse	1464	6.27%
Physician	428	1.83%
Patient	248	1.06%
Other	241	1.03%
Medication Error Categories		
Category A	7344	31.45%
Category B	13,150	56.30%
Category C	1099	4.71%
Category D	1741	7.45%
Category E	2	0.01%
Category F	5	0.02%
Not Specified	14	0.06%
Medication Error Stages		
Prescribing	16,481	70.57%
Preparing	1702	7.29%
Administration	1576	6.75%
Dispensing	1513	6.48%
Transcribing	1150	4.92%
Monitoring	933	3.99%

**Table 2:** Reported medications errors classified by USP therapeutic categories.

Therapeutic Category	Frequency (n)	Percentage (%)
TOTAL (n) = 23,355		
Central Nervous System Agents	13,076	55.99%
Antipsychotics	7769	59.41%
Antidepressants	3829	29.28%
Anticonvulsants	750	5.74%
Sedatives/Hypnotics	607	4.64%
Nicotine dependence	46	0.35%
CNS stimulants and ADHD	38	0.29%
Antiparkinsonian Agents	37	0.28%
Cardiovascular Agents	3745	16.04%
Various	2319	9.93%
Endocrine Agents	1764	7.55%
Gastrointestinal Agents	1525	6.53%
Mineral Supplements	559	2.39%
Antimicrobial Agents	367	1.57%

**Table 3:** Reported medication errors for the top twenty medications.

Medications	Frequency (n)	Percentage (%)
TOTAL (*n*) = 23,355		
Olanzapine	1650	7.06%
Omeprazole	1350	5.78%
Quetiapine	1273	5.45%
Mirtazapine	1120	4.80%
Haloperidol	1045	4.47%
Risperidone	1000	4.28%
Nifedipine	984	4.21%
Escitalopram	915	3.92%
Glibenclamide	910	3.90%
Benzhexol Hydrochloride	797	3.41%
Sodium Valproate	727	3.11%
Atorvastatin	665	2.85%
Labetalol	609	2.61%
Lorazepam	458	1.96%
Clozapine	377	1.61%
Aripiprazole	370	1.58%
Fluoxetine	364	1.56%
Insulin	354	1.52%
Lithium Carbonate	352	1.51%
Chlorpromazine Hydrochloride	350	1.50%

## Data Availability

The data are not publicly available due to privacy or ethical restrictions.
